# Dewetting behavior of Ge layers on SiO_2_ under annealing

**DOI:** 10.1038/s41598-020-70723-6

**Published:** 2020-08-13

**Authors:** A. A. Shklyaev, A. V. Latyshev

**Affiliations:** grid.415877.8 0000 0001 2254 1834 A.V. Rzhanov Institute of Semiconductor Physics, SB RAS, Novosibirsk, 630090 Russia

**Keywords:** Materials science, Nanoscience and technology

## Abstract

The solid-state dewetting phenomenon in Ge layers on SiO_2_ is investigated as a function of layer thickness *d*_Ge_ (from 10 to 86 nm) and annealing temperature. The dewetting is initiated at about 580–700 °C, depending on *d*_Ge_, through the appearance of surface undulation leading to the particle formation and the rupture of Ge layers by narrow channels or rounded holes in the layers with the thicknesses of 10–60 and 86 nm, respectively. The channel widths are significantly narrower than the distance between the particles that causes the formation of thinned Ge layer areas between particles at the middle dewetting stage. The thinned areas are then agglomerated into particles of smaller sizes, leading to the bimodal distributions of the Ge particles which are different in shape and size. The existence of a maximum in the particle pair correlation functions, along with the quadratic dependence of the corresponding particle spacing on *d*_Ge_, may indicate the spinodal mechanism of the dewetting in the case of relatively thin Ge layers. Despite the fact that the particle shape, during the solid-state dewetting, is not thermodynamically equilibrium, the use of the Young’s equation and contact angles allows us to estimate the particle/substrate interface energy.

## Introduction

The solid-state dewetting phenomenon can be used for the development of the simple and highly productive fabrication methods of dense nano- and micro-sized particle arrays^1–6^. The metal particles obtained by dewetting are widely used due to their ability of surface plasmon generation^7–9^. At the same time, the submicron- and micron-sized dielectric particles, when interacting with light, generate magnetic and electrical resonances. The strong magnetic dipole resonance is excited in particles of materials with a high refractive index (*n*) under the condition *λ≈nd*, where *λ* is the incident light wavelength and *d* is the particle size^10^. In this aspect, Si submicron structures are widely studied. Taking into account that the refractive index of Ge is greater than that of Si and the spectral dependence of their light absorption coefficients, Ge appears as a promising material for electromagnetic resonance engineering in the near infrared spectral range^11–15^. The Ge particle formation can occur by means of the solid-state dewetting phenomenon during annealing of Ge layers on SiO_2_. The particles are formed spontaneously and acquire broad distributions in shape and size. The surfaces coated with such particles exhibit strong antireflection properties in a wide spectral range^16^. This initiates the study of light scattering by quasi-random surface structures which can also be formed by other methods^17–20^.

Initially, the study of the Ge particle formation on SiO_2_ surfaces was aimed at obtaining nanometer-sized three-dimensional Ge islands in the range from several to about 40 nm, which were considered to serve as quantum dots^21–28^. It was shown that Ge islands can be formed at relatively low temperatures, i.e. after annealings at *T* ≥ 275 °C of the samples with a Ge layer grown on SiO_2_ at room temperature^29^, or during the Ge deposition at *T* ≥ 320 °C by the molecular-beam technique^23^. The possibility of obtaining pure Ge islands (particles) of larger sizes, according to our knowledge, has not been investigated, whereas the submicron- and micron-sized dielectric particles are required for the photonic application in the visible and infrared spectral ranges. Rather large silicon-rich Ge particles (100–200 nm in the lateral dimension) can be created by means of annealing strained Ge/Si/SiO_2_/Si structures at 700 °C^30^. Recently, the large-sized SiGe particles have been grown by the relatively thick Ge layer (~ 40 nm) deposition at 800 °C on Si/SiO_2_^16^. When using thick continuous Ge layers on SiO_2_ for their subsequent annealing at relatively low temperatures, the dewetting driving force may be insufficient for the layer agglomeration into arrays of individual particles. This effect is known for metal layers on dewetting surfaces, for which it was found that the minimum temperature necessary for the layer agglomeration is higher for thicker metal layers^1,31,32^. The thickness dependence of the dewetting process of Ge layers on SiO_2_ is less studied. Cheynis et al. showed that the time for completing the dewetting process increases with the thickness in the 10–40 nm range at a given temperature of about 800 °C^33^. It was also found that the dewetting behavior of 60-nm Ge layers depends on the Ge deposition method^34^.

The Ge particle formation on SiO_2_ can be carried out in two ways. This can occur during the Ge deposition if the substrate temperature is sufficiently high^21,35–37^. Ge particles can also be formed as a result of the high-temperature annealing of continuous Ge layers grown on SiO_2_ at relatively low temperatures^22,24,29,33,34^. The second approach gives greater opportunities, since it allows avoiding the SiO_2_ surface etching during the Ge deposition, which occurs via the formation of volatile SiO and GeO molecules^21,38^. The annealing of grown structures can be performed directly in the growth chamber or after the sample removal into the air. In the latter case, the Ge layer surface is covered with a native oxide, which can interfere with the subsequent dewetting process^39^ requiring a higher temperature for its initiation than in the first case. However, placing the sample in the air can happen for technological reasons, for example, for carrying out lithography. It is reasonable to assume that the native Ge oxide, similar to the chemically grown Si oxide^39^, can affect the minimum temperature for the dewetting process activation.

In this study, in order to obtain submicron-sized Ge particles, we use rather thick (from 10 to 86 nm) continuous Ge layers initially deposited on SiO_2_ at room temperature (RT) for a subsequent high-temperature annealing. As expected, the layers are thermally unstable and agglomerated into compact particles at 600 °C and higher temperatures depending on their thickness (*d*_Ge_). Their agglomeration into particles occurs through the solid-state dewetting. The features of the process indicate that at *d*_Ge_ of 10–40 nm, it proceedes by the spinodal mechanism, which led to the formation of a homogeneous and dense array of submicron-sized Ge particles. The agglomeration of thicker Ge layers occurs non-uniformly over the surface and lead to the formation of particles, which differ in size and shape, with a bimodal distribution. An analysis of the shape of Ge particles shows that their large values of the contact angles and aspect ratios (height of particles divided by their lateral dimension) are the result of the excess of the Ge/SiO_2_ interface energy over the SiO_2_ surface energy. Our study determines the conditions of obtaining the surface coatings of submicron-sized dielectric Ge particles using the dewetting phenomenon depending on *d*_Ge_ and annealing temperature (*T*_ann_).

## Results

### Behavior of 10–40 nm Ge layers on SiO_2_ under annealing

**Figure 1:**
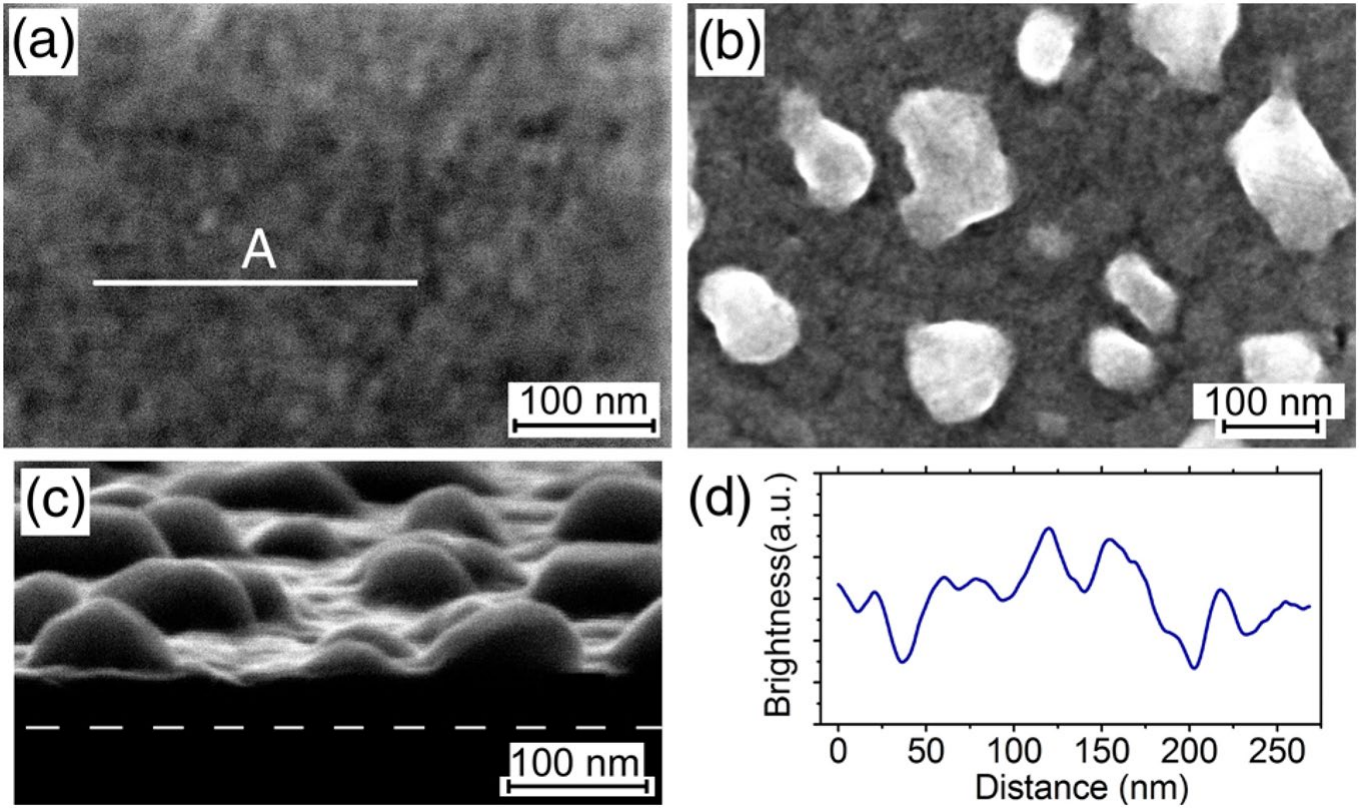
(**a**–**c**) SEM images of 20, 86 and 60 nm Ge layers deposited on SiO_2_ at RT with a subsequent annealing in vacuum for 30 min at 560, 700 and 650 °C, respectively. (**d**) The brightness profile along line A is shown in (**a**). The white dashed line in (**c**) shows the position of the Ge/SiO_2_ interface, which is not visible due to the increased image contract. Images (**a**,**b**) are taken at the normal electron beam incidence and (**c**) at a glancing angle of about 10° relative to the sample surface.

It was shown that low-temperature (up to 200 °C) Ge grown on SiO_2_ forms polycrystalline Ge layers^40^, which remain continuous and exhibit a high hole mobility after the annealing at temperatures up to 500 °C^41^. The annealing of our samples at 550 °C and lower temperatures does not lead to the formation of three-dimensional particles. The changes in the surface morphology are observed after the annealing at temperatures higher than 550 °C (Fig. 1). The changes occur through the appearance of undulations on the surface of Ge layers. This becomes visible in the SEM images^42^ due to the difference in the atomic number between Ge from one side and, Si and oxygen from the other, at which the surface areas with thicker Ge layers on SiO_2_ look brighter (Fig. 1d). In the case of the thinner (~ 20 nm) Ge layers, the distance between protuberances (Fig. 1a) is significantly smaller in comparison with that for the thicker Ge layers (Fig. 1b,c). Since the Ge layers are polycrystalline, their surface consists of areas with different crystalline orientations^41^. Although we do not know the size distribution of grains and their orientation with respect to the substrate surface, it is reasonable to assume that surface areas with grains with certain crystalline orientations may grow due to the Ge consumption from other areas. This may explain the appearance of protuberances on the surface at the relatively low annealing temperatures.

**Figure 2:**
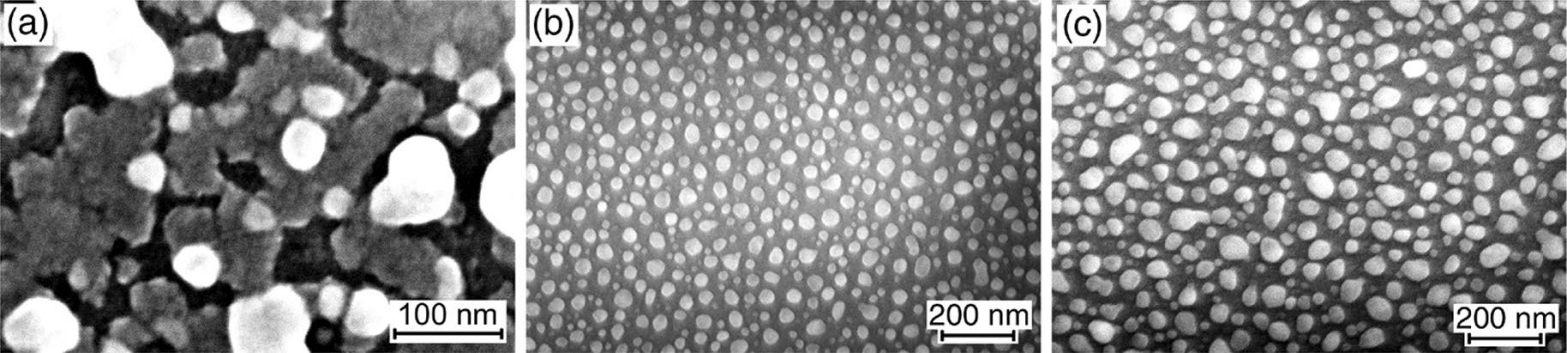
SEM images of Ge layers deposited on SiO_2_ at RT with a subsequent annealing in vacuum after the exposure to the air: (**a**) 20 nm Ge layer annealed at 580 °C, (**b**) and (**c**) 10 and 20 nm Ge layers, respectively, annealed at 600 °C.

After the annealing at 580 °C for 30 min, the partial agglomeration of 10 and 20 nm Ge layers is observed. It consists in the formation of relatively large particles near holes shaped as narrow channels and small particles atop the remaining areas of the continuous Ge layer, as shown in Fig. 2a. Longer anneals (~ 2 h) at 580 °C do not lead to the complete disappearance of remaining areas of the Ge layers between the particles. The annealing at 600 °C ensures complete agglomeration of the Ge layers into compact particles (Fig. 2b). However, their average size is smaller than that of the particles after the annealing at 580 °C. This indicates that the particle formation mechanisms are different at the relatively low and high temperatures. The annealing at 800 °C, compared with the annealing at 600 °C, results in the formation of more compact particles with a more uniform shape, whereas their concentration and average size do not differ significantly.

**Figure 3:**
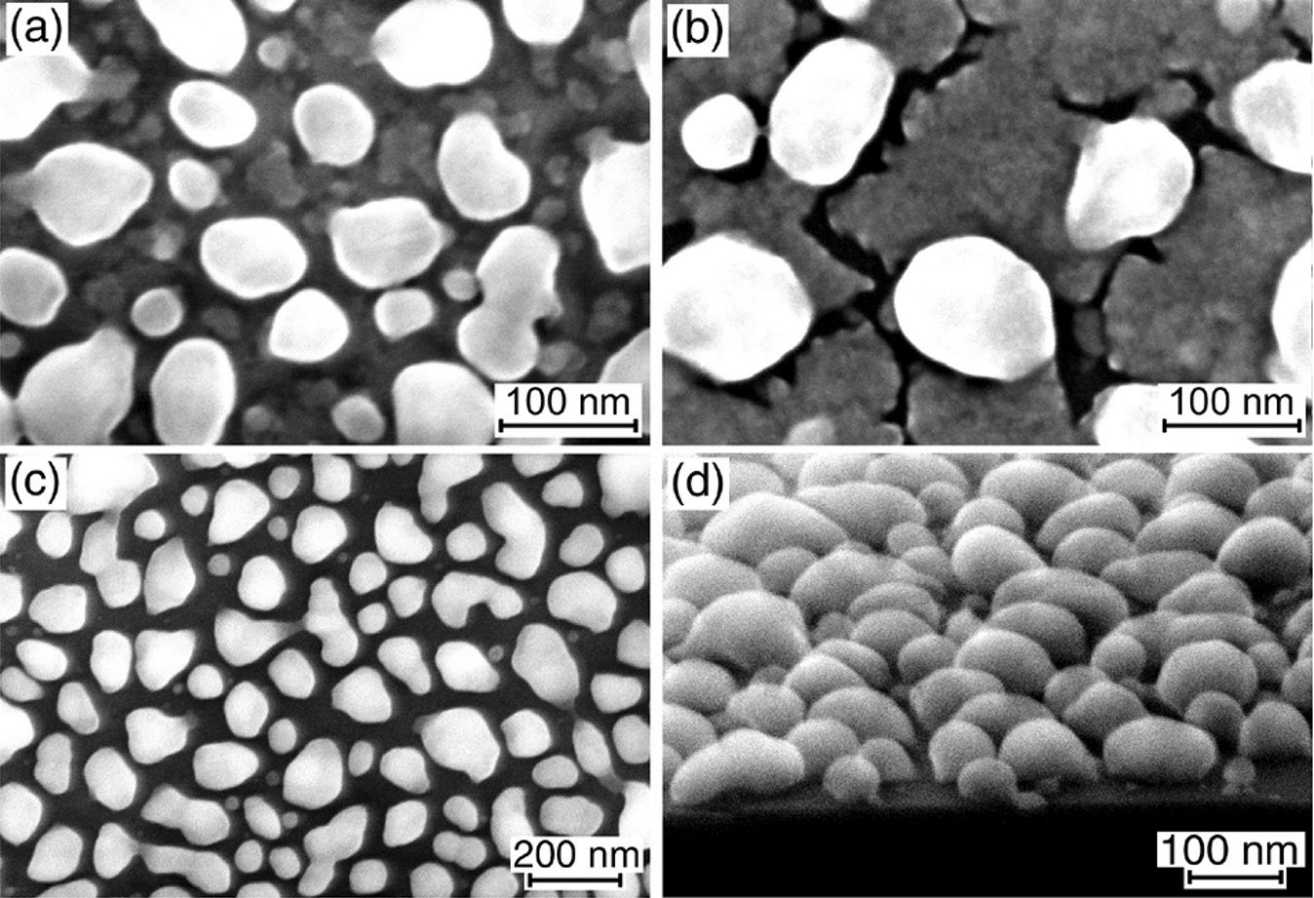
SEM images of the samples covered with (**a**) 30 and (**b**) 40 nm Ge layers after the annealing at 600 °C and (**c**) with the 40 nm Ge layer after the annealing at 800 °C. The image in (**d**) is taken at a glancing angle of about 10° for the sample with the 40 nm Ge layer after the annealing at 700 °C.

The agglomeration of 30 and 40 nm Ge layers at 600 °C is partial (Fig. 3a,b). The dewetting proceeds through the development of large thickness perturbation of the Ge layers and the simultaneous formation of narrow channels (Fig. 3b). The ratio between these two processes also has a strong dependence on the annealing temperature. The remaining areas of the Ge layers between Ge particles completely disappear after the annealing of 30 and 40 nm Ge layers at 700 and 800 °C (Fig. 3c,d), respectively, and the particles acquires a compact shape with aspect ratios of about 0.5 (Fig. 3d). It can be noted that higher annealing temperatures are usually required to initiate the dewetting process in thicker layers of many materials^1,43^, including Si on SiO_2_^2,39^.

The protuberances on the surface after the annealing at relatively low temperatures, the same as grains of the initial polycrystalline Ge layers, have a large scatter in size and random distribution over the surface^41^. Therefore, the subsequent appearance of channels in thinned areas of the Ge layers can be associated with grain boundaries. In contrast, after annealing at the high temperatures, the concentration of Ge particles is much higher than that of the protuberances, and they are uniformly distributed over the surface. This may indicate that their formation is not related to the location of grain boundaries. In addition, the Ge particle concentration gradually increases with the increasing *d*_Ge_, while such dependence for the grain concentration has not observed^41^.

### Dewetting behavior of 60–86 nm Ge layers

**Figure 4:**
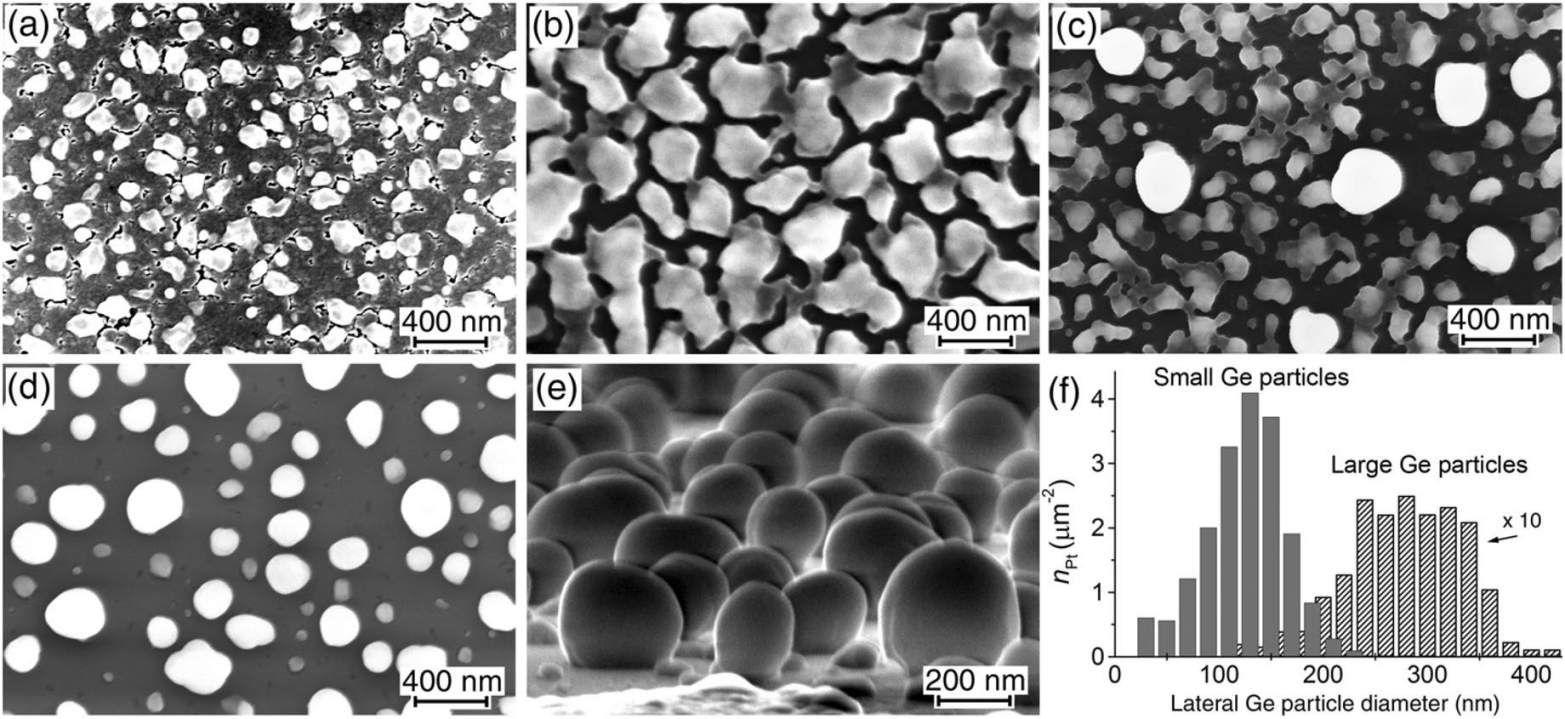
SEM images of the samples with the 60 nm Ge layers after the annealing at (**a**) 650, (**b**) 700, (**c**) 800 and (**d**,**e**) 850 °C. Images (**a**–**d**) are taken at the normal electron beam incidence and (**c**) at the glancing angle of about 7° relative to the sample surface. (**f**) The bimodal distribution of Ge particles on SiO_2_ as a function of their lateral diameter, which is obtained for the sample with the 60 nm Ge layer after its annealing at 800 °C.

The surface morphology of the samples with 60 and 70 nm Ge layers exhibits a strong dependence on *T*_ann_, as shown in Fig. 4a–e. The annealing at 650 °C results in the surface morphology (Fig. 4a) similar to that observed for the 40 nm Ge layers after 600 °C. However, the annealing at 800 °C leads to a significantly different morphology. It consists in the formation of a bimodal distribution of Ge particles which are different in size and shape (Fig. 4c,f). This arises from the specific features of the Ge layer evolution under annealing. It begins from the formation of large Ge particles and areas of the thinned Ge layer between them. These areas then turn into groups of smaller Ge particles after the annealing at 850 °C (Fig. 4d). Some of the Ge particles acquire a shape close to spherical with the aspect ratio of about 1, as shown in Fig. 4e.

The longer annealing (~ 2 h) at temperatures up to 800 °C results only in slight surface morphology changes making the Ge particles more compact. This indicates that the main changes in the surface morphology at each temperature occur during the initial period of annealing time. Then, the rate of change decreases as the total energy of surface layers approaches its minimum. This suggests that the surface morphology can depend on the rate at which the annealing temperature is reached. However, in this study, only the rather high rate of 4 °C/s is used.

**Figure 5:**
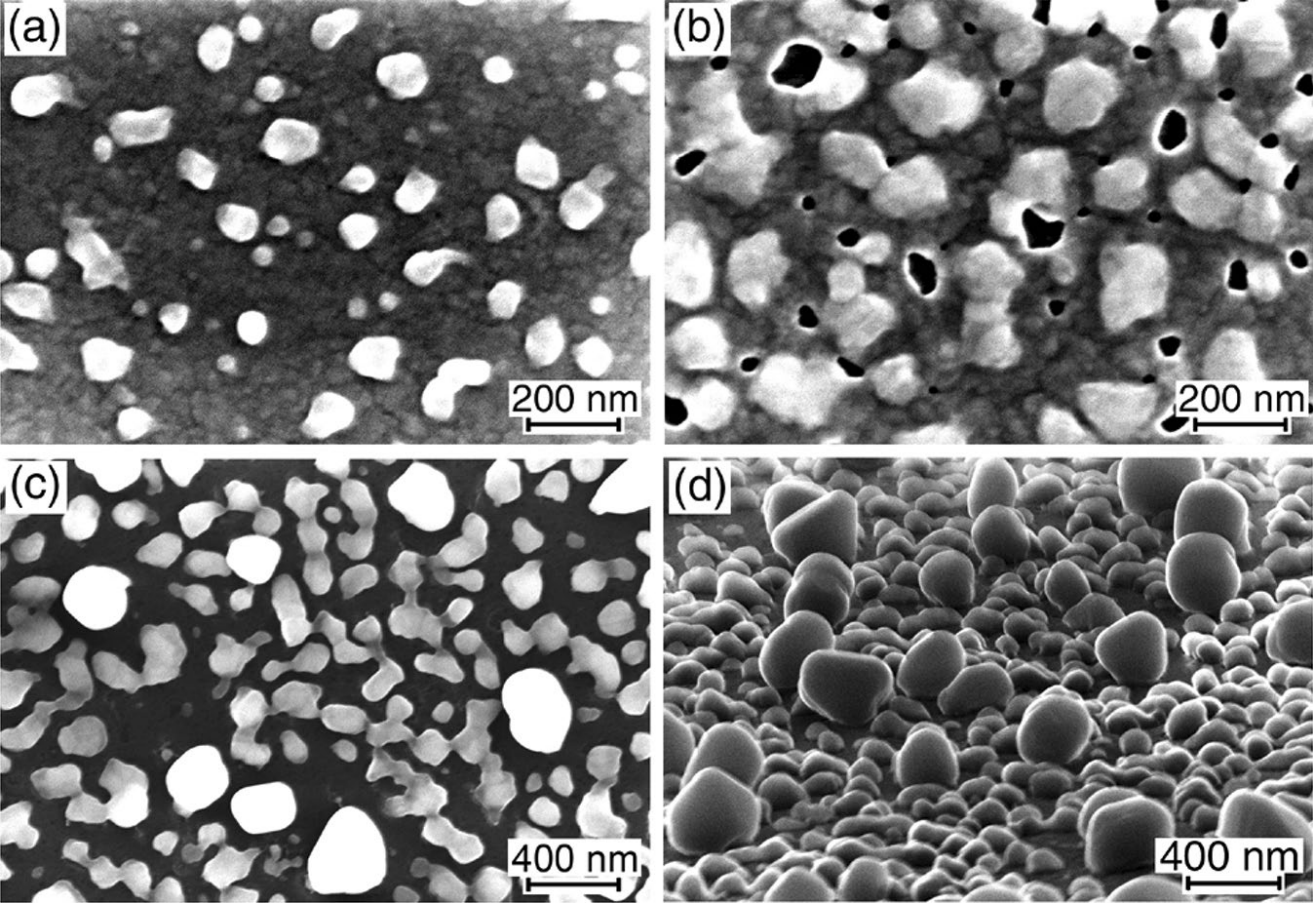
SEM images of the samples with 86 nm Ge layers on SiO_2_ after the annealing at (**a**) 700, (**b**) 800 and (**c**,**d**) 850 °C. Images (**a**–**c**) are taken at the normal electron beam incidence and (d) at a glancing angle of about 10° relative to the sample surface.

The break-up behavior of 86 nm Ge layers at the initial stage under the annealing is similar to that of thinner Ge layers. At 700 °C, this consists in the formation of Ge protrusions like three-dimensional particles only due to the thinning of the surrounding Ge layer (Figs. 1b, 5a), that is, without the appearance of holes shaped as channels. This may be interpreted as the Ge layer thickness undulation. As was mentioned for the initial stage of thin Ge layer transformation, the undulation formation can be associated with different crystalline orientations of Ge grains in the Ge layers, which may have different growth rates during annealing.

The break-up development at the high temperature of 800 °C occurs through the compact hole formation in the Ge layer (Fig. 5b). After the annealing at 850 °C, the formation of Ge particles with bimodal distributions in size and shape is observed (Fig. 5c,d), similar to the behavior of 60 and 70 nm Ge layers after the annealing at 800 °C. The compact particles of larger sizes are characterized by a large aspect ratio up to about 1.0 (Fig. 5d). It can be mentioned that the dewetting in Si on SiO_2_ results in the formation of Si particles with significantly smaller aspect ratios up to 0.4^44,45^.

The formation of holes, as well as channels during the annealing of thinner Ge layers is aimed at the exposing of SiO_2_ surface areas. This occurs for the surface energy minimization, since the SiO_2_ surface energy is much smaller than that of Ge.

### Surface morphology after the annealing at 900 °C

**Figure 6:**
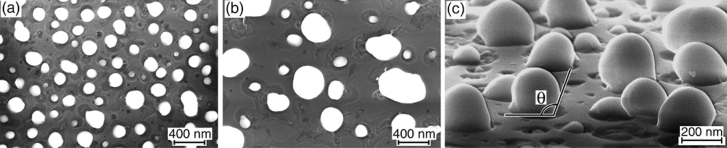
(**a**–**c**) SEM images of the samples with 40, 70 and 60 nm Ge layers on SiO_2_ after the annealing at 900 °C for 30 min. Images (**a**,**b**) are taken at the normal electron beam incidence and (**c**) at a glancing angle of about 5° relative to the sample surface. The particle contact angle is shown in (**c**).

The annealing of 40–86 nm Ge layers at 900 °C lead to the formation of compact Ge particles, the shape of which becomes more uniform (Fig. 6). As expected, a certain Ge amount is removed from the surface due to the annealing. This process was studied earlier^21,38,46^ and consisted in the formation of volatile SiO and GeO molecules by the reaction

1 $$ {\text{SiO}}_{{2}} + {\text{ Ge}} \to {\text{SiO}} \uparrow + {\text{ GeO}} \uparrow , $$

occuring at the boundary between SiO_2_ and Ge particles along their perimeter. This is confirmed by the fact that voids (pits) are formed in SiO_2_ around relatively large Ge particles, and they also appears at the locations of small Ge particles after their complete removal (Fig. 6). The similar result was observed for SiGe-on-SiO_2_ structures with strained SiGe layers after their high-temperature annealing at 880 °C^47^. The interaction of Si particles with SiO_2_ wa**s** previously studied in detail^48–50^, and it revealed the features similar to those observed in our case.

### Discussion

**Figure 7:**
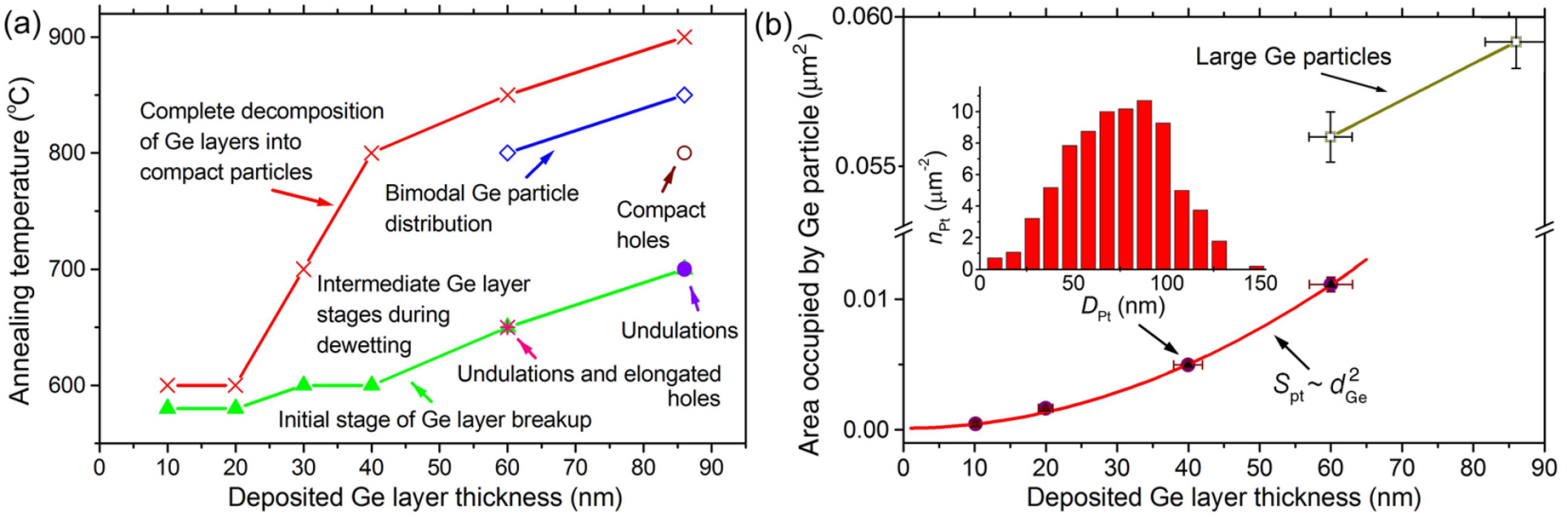
(**a**) Schematic diagram of Ge layer transformations on SiO_2_ caused by dewetting, presented in coordinates *d*_Ge_ and *T*_ann_. (**b**) Average surface area occupied by a Ge particle after the dewetting of Ge layers from SiO_2_ as a function of *d*_Ge_. The annealing of the deposited Ge layers is performed at 800 °C for 30 min for the layer thicknesses of 10, 20 40 and 60 nm, and at 850 °C for 86 nm. The concentration Ge particle distribution as a function of their lateral diameter, obtained after the dewetting of the 40 nm Ge layer, is shown in the inset.

A general picture of the Ge layer transformations caused by the dewetting from the SiO_2_ surface is schematically shown in Fig. 7 in coordinates *d*_Ge_ and *T*_ann_. The behavior of 10 nm Ge layers on SiO_2_ under annealing was previously investigated by Wakayama et al.^29^. They observed the Ge particle formation at about 300 °C when it was carried out in a vacuum chamber without the sample exposition to the air. The significantly higher temperatures require for the Ge particle formation, in our case, can be associated with the presence of the native Ge oxide, which can interfere with the dewetting process^39^. The beginning of the Ge particle formation may indicate that the stabilizing effect of the native Ge oxide becomes insufficient at 580 °C. Probably, at this temperature, its partial decomposition occurs with the formation of volatile GeO molecules. This is consistent with the fact that the onset of the dewetting process is observed at about the same temperatures around 600 °C for the Ge layers in the wide range of *d*_Ge_ (Fig. 7a). The GeO_2_ surface energy is less than that of SiO_2_^51^, and it is much smaller than the Ge surface energy. Therefore, the Ge layers covered with the native Ge oxide are thermally stable. Conditions for their agglomeration into particles appear when this Ge oxide begins to be desorbed with the formation of bare Ge surface areas. The similar effect of the native oxide could also manifest itself in the behaviour of thin crystalline Si layers on SiO_2_, for which the Si particle formation was observed after the annealing at significantly higher temperatures (> 750 °C) for commercial silicon-on-insulator structures^39,52–54^ than that (*T*_ann_ = 550 °C) for the bare amorphous Si layers on SiO_2_^29^.

One of the approaches to obtaining clean Ge surfaces consists in an ex situ wet etching/oxidation process which provides removal of metallic and carbon contaminations. This is followed by in situ thermal annealing in vacuum at 500–600 °C^55,56^ or flash anneals at about 750 °C^57,58^ to remove Ge oxide. In our case, the initial surface of deposited Ge layers does not contain impurities. Therefore, the onset of the dewetting process, which is observed at 600 °C, can be associated with the removal of native oxide from the Ge surface.

The averaged lateral area (*S*_pt_) occupied by a Ge particle increases proportionally to ~ $d_{Ge}^{2}$ (Fig. 7b). A typical concentration distribution (*n*_pt_) of compact Ge particles as a function of their diameter is shown in the inset to Fig. 7b. The obtained relation *S*_pt_ ~ $d_{Ge}^{2}$ means that *D*_pt_ ~ *d*_Ge_, where *D*_pt_ is the average particle diameter. A linear relation between *D*_pt_ and *d*_Ge_ is usually observed when metal particles are formed by dewetting^1,31^. Such a relation between *D*_pt_ and *d*_Ge_ corresponds to the decrease in the total particle concentration *N*_pt_ as a function of *d*_Ge_ as *N*_pt_ ~ 1/$d_{Ge}^{2}$^43^. The strong decrease of *N*_pt_ as a function of increasing *d*_Ge_ also occurs during the Ge deposition on Si(100)^5^.

There are two mechanisms of the dewetting phenomenon, which are usually distinguished in thin layers deposited on non-wettable surfaces. One of them is called spinodal dewetting, which is associated with a spontaneous amplification of layer thickness perturbation^6,59^. The spontaneous growth of the perturbation can eventually reach the substrate surface. Dewetting can also proceed through the hole formation, which begins at the inhomogeneities in the layer surface or layer/substrate interface^6,51^. In our case, as discussed below, the first or both dewetting mechanisms are involved, depending on *d*_Ge_ and *T*_ann_.

At the relatively high temperatures the compact Ge particle formation is accompanied by the rupture of continuous Ge layers with the appearance of holes shaped as narrow 10–30 nm wide channels (Fig. 1b). The channel width is approximately equal to the distance between the Ge particles arising from the agglomeration of 10 and 20 nm Ge layers. This results in the narrow temperature range (~ 580 to 600 °C) from the initial agglomeration stage to the formation of compact Ge particles (Figs. 2, 7a). However, the channel width is smaller than the distance between Ge particles (protrusions) in the case of the thicker (30–60 nm) Ge layers. This lead to the intermediate presence of thinned areas of the Ge layers near the Ge protrusions and particles. The further thinning of the thinned areas makes them thermally unstable and causes their agglomeration into the groups of relatively small particles (Figs. 3, 4, 5).

**Figure 8:**
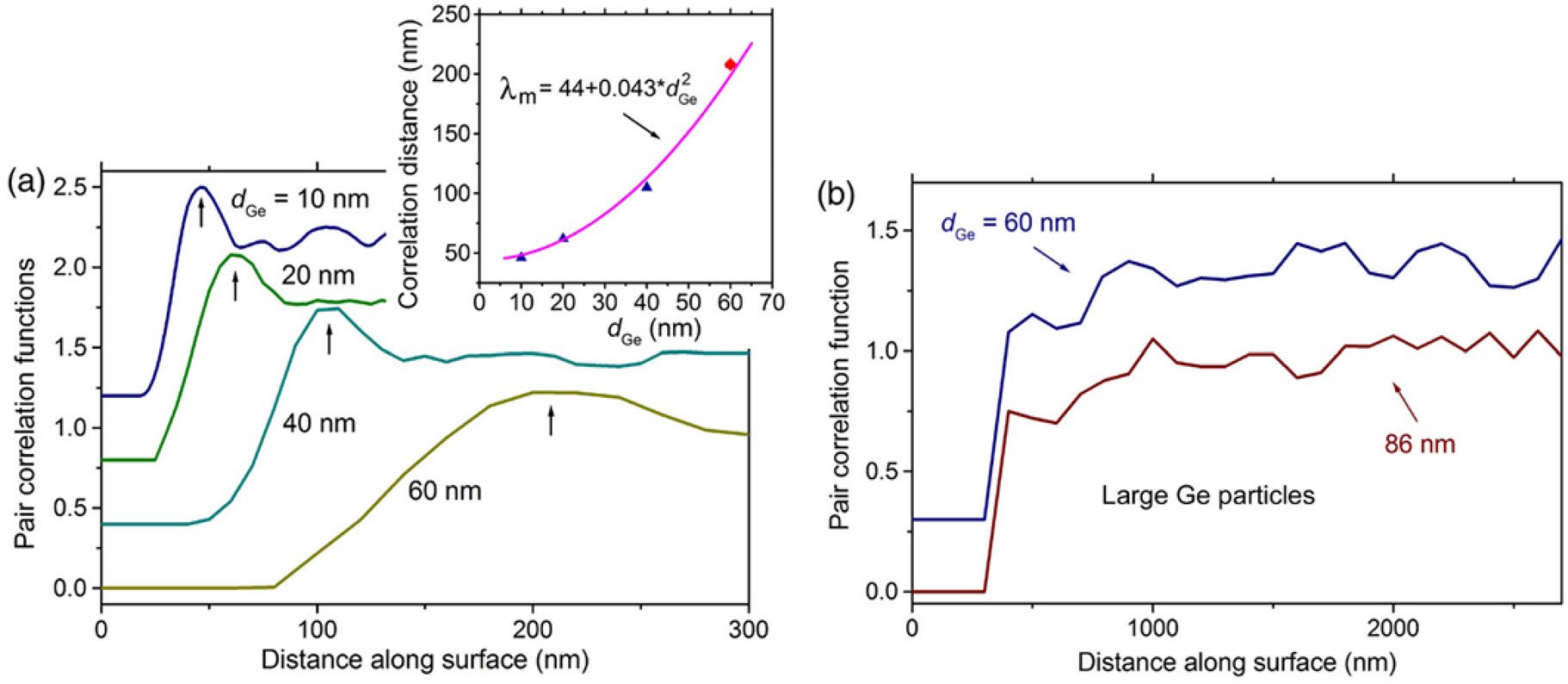
Pair correlation functions of the mass centers of the Ge particles on SiO_2_ obtained after the dewetting of relatively (**a**) thin and (**b**) thick Ge layers. The Ge layer thicknesses are marked at the corresponding curves. The data for the 60 nm Ge layer in (**a**) are obtained without taking into account large Ge particles. The functions are offset from each other by 0.4 on (**a**) and by 0.6 on (**b**) for their separation. The pair correlation length (*λ*_m_), which positions are marked in (**a**) by arrows, as a function of the deposited Ge layer thickness is shown in the inset.

The Ge particles form a dense array for which a short-range order in the Ge particle spatial distribution can be expected. To reveal this, we calculate the pair correlation function of the mass centers of Ge particles (Fig. 8). For the case of 10–60 nm Ge layers, the pair correlation functions exhibit well pronounced maxima (Fig. 8a) at the preferable particles spacing (*λ*_m_). The presence of the maxima is the feature of the spinodal mechanism of the dewetting process^60,61^. The other feature of spinodal dewetting is the quadratic dependence of *λ*_m_ on the thickness of deposited layers^61–64^. In our case, such dependence looks as *λ*_m_(nm) *≈* 44(nm) + 0.043(nm^−1^) × $d_{Ge}^{2}$ (the inset in Fig. 8a). It shows that as *d*_Ge_ → 0, *L* does not → 0, as would be expected. This deviation from the typical behavior may indicate the influence of native Ge oxide residues on the layer thickness undulation at the initial dewetting stage. This may also reflect the influence of polycrystalline nature of the Ge layers, if the size of Ge grains does not tend to 0 with the decreasing Ge layer thickness.

Maxima are not observed in the pair correlation functions of large Ge particles which are formed using 60 and 86 nm Ge layers (Fig. 8b). The distance between them is so great that they do not influence each other. This indicates that the large Ge particle formation mechanism cannot be attributed to the spinodal one. This is despite the fact that the gradual change in the surface morphology is observed through the formation of protrusions without holes at the initial stage (Figs. 1b, 5a). As mentioned above, their formation can be associated with the surface areas of initial Ge grains with certain crystalline orientations.

In the case of liquid-state dewetting, the shape of droplets (particles) is characterized by the contact angle *θ* which is described by the Young’s equation^1,65^. This can be written in the form:

2 $$ \gamma_{is} = \gamma_{ss} - \gamma_{ps} \cos (\theta ) $$

where $\gamma_{is}$ is the particle/substrate interfacial tension; $\gamma_{ss}$ and $\gamma_{ps}$ are the surface tensions of the substrate and particles, respectively. It is important to note that Eq. (2) is valid for describing thermodynamically equilibrium particle shapes. In the case of solid-state dewetting, the particle shape is determined by kinetic factors, such as nucleation of crystal particles with an arbitrary orientation relative to a substrate surface and growth rates which depend on the crystalline orientation of particle surface planes. This forms particles which can differ in shape. However, if the particle formation conditions are not far from equilibrium, their shape can be characterized by Eq. (2) using surface and interface energies instead of tensions^66,67^. In our case, some scatter of *θ* can be seen in Fig. 6c even when the high *T*_ann_ (900 °C) is used. This means that the particle formation conditions are not very close to equilibrium. Nevertheless, Eq. (2) can be used for the semi-quantitative estimates of the energy parameters of surface structures.

Assuming that the surface energies of amorphous SiO_2_ and Ge are known, we can use Eq. (2) to determine the interface energy between the Ge particles and SiO_2_ as a function of the contact angle. The surface energy of Ge ranges from 1.32 to 1.71 J/m^2^, depending on the crystalline orientation of its surface^68,69^. $\gamma_{fs}$ = 1.5 J/m^2^ can be taken as an average value. The surface energy $\gamma_{ss}$ of SiO_2_ can be determined through the contact angles of droplets (particles), which are formed due to the dewetting of various liquids on SiO_2_^70,71^, including melted Ge^34^. This gives $\gamma_{ss}$** ~ **0.05 J/m^2^. The SiO_2_ surface energy obtained by calculations and other experimental methods exhibits much larger values $\gamma_{ss}$**~** 0.4 J/m^2^^72–74^. As was shown by Bangera and Appaiah^75^, this is a typical relation between of the surface energy values when they are smaller by a factor of ~ 10 if they are determined in the liquid-state dewetting experiments. Just as it was done by Cheynis et al.^33^ in their analysis of the solid-state dewetting, the larger values of $\gamma_{ss}$, namely ≈ 0.4 J/m^2^, should be used in our estimations of $\gamma_{is}$. In Fig. 6c are the SEM images of Ge particles with *θ* ranges from about 90° to 120° which are formed even around one particle. Taking *θ* = 105° as an average value, the Eq. (2) gives $\gamma_{is}$ ≈ 0.8 J/m^2^. This value is consistent with the literature data, according to which the Ge/SiO_2_ interface energy is larger than the SiO_2_ surface energy^73,74^. In the structures with such relationships between $\gamma_{is}$ and $\gamma_{ss}$, the size of the base of particles should be smaller than their lateral dimension. For comparison, in the structures of SiGe particles on Si surfaces, *θ* < 90° and, hence, the SiGe/Si $\gamma_{is}$ of is smaller than the Si $\gamma_{ss}$^5^. This may indicate that a larger *θ* may be associated with a larger driving force for dewetting. This is consistent with the fact that the dewetting in Ge/SiO_2_ is characterized by larger *θ* values and occurs at a lower temperature in comparison with Ge/Si which requires higher temperatures to intiate the dewetting and leads to smaller *θ* values^5^. It can be noted that there is an estimation of the driving force for the dewetting in Si on SiO_2_ structures, which resulted in 14 eV/nm^2^^2^.

The surface morphology evolution due to dewetting can occur simultaneously over the entire surface. This was observed in the structures such as Ge/Si(111)^42,76^ and Ge/SiO_2_^29,33,34^. If the driving force for dewetting is relatively week and the surface morphology transformation begins from defects, the dewetting develops through a gradual propagation along the surface. Such behavior takes place in the structures of Ge/Si(100)^77–79^ and Si(crystalline)/SiO_2_^3,41,80–82^. The dewetting conditions may appear in the structure with the increasing elastic strain in it, as was observed when the thickness of Ge layers on Si(111) was increased at a given substrate temperature^83^. The surface layer energy calculations for the Ge/Si(100) structures showed that it has a gentle minimum as a function of the contact angle. This was proposed as an argument for explaining the large scatter of contact angles^5^. In our case of polycrystalline Ge layers up to 60 nm thick grown on SiO_2_ at RT, the dewetting phenomenon occurred simultaneously over the entire surface with the formation of Ge particles in the shapes with a relatively small scatter of contact angles. This indicates a relatively large driving force for the dewetting in Ge/SiO_2_ relative to Si/SiO_2_.

### Conclusion

The initiation of dewetting in the 10–86 nm Ge layers on SiO_2_ after the exposure to the air begins at temperatures of 580–700 °C, depending on the layer thickness. At the relatively low temperatures, this occurs through the appearance of undulation on the surface of Ge layers, which can be associated with their polycrystalline structure containing grains with different crystalline orientations. Then, the formation of Ge particles is accompanied by a rupture of the Ge layers with the appearance of narrow channels or rounded holes in the Ge layers with the thicknesses of 10–60 and 86 nm, respectively. The distance between particles is comparable in magnitude with the channel widths in the 10–20 nm Ge layers. The formation of compact Ge particles in this case is completed at 600 °C. For the 30–86 nm Ge layers, the width of the channels or holes is less than the distance between the particles. As a result, along with them, the thinned Ge layer areas are formed at the intermediate break-up stage. As these areas are thinned further, they break up into smaller particles. This leads to the bimodal distribution of particles which differ both in size and in shape. Such features as the existence of a maximum in the particle pair correlation functions and the quadratic dependence of the corresponding particle spacing on *d*_Ge_, may indicate the spinodal dewetting mechanism for the formation of small particles at the relatively high temperatures. The larger particles, which begin to form first, acquire a high aspect ratio up to about 1.0. The contact angles of the Ge particles are larger than 90°, thus, indicating that the interface energy between the Ge particle and SiO_2_ is greater than the SiO_2_ surface energy.

## Methods

### Sample preparation

The Si(100) plates covered with a 1 μm thick thermal SiO_2_ film are used as substrates, the same as in^34^. The Ge deposition is carried out in an ultrahigh-vacuum chamber, with a base pressure of about 1 × 10^–10^ Torr, which was manufactured by Omicron. A Knudsen cell with a BN crucible is used for the Ge deposition at a rate of ~ 1.0 nm/min. The Ge deposition rate was calibrated as a function of Knudsen cell temperature by means of measuring submonolayer Ge coverages on Si(111) with scanning tunneling microscopy^84^. The accuracy of setting the Ge layer thickness is determined by the accuracy of measuring the Ge deposition rate, which is within 5%. After the substrate degassing by heating in the growth chamber at about 450 °C, the Ge deposition is performed without substrate annealing. During the Ge deposition the substrate temperature may gradually increase from RT due to the radiation from the Knudsen cell. The substrates covered with Ge layers from 10 to 86 nm thick are cut into samples, which are then annealed in the vacuum conditions of a MILA-5000 furnace with a residual pressure of ~ 1 × 10^–7^ Torr at various temperatures up to 900 °C for 30 or 120 min. The heating and cooling temperature rates are about 4 and 15 °C/s, respectively.

### Material characterization

The sample surface morphology is studied using a Pioneer scanning electron microscope (SEM) manufactured by Raith. The sample surface observation is performed at different electron beam incident angles, including obtaining the images of sample cross-sections, similar to that used elsewhere^34^. All SEM images presented here were taken from the samples after their annealing for 30 min. The average value of areas occupied by a Ge particle is obtained from SEM images using Gwyddion software. The spread of this value between surface areas is less than 5%.
